# Safety and immunogenicity of 13-Valent pneumococcal conjugate vaccine in healthy infants in Shanghai during the COVID-19 pandemic: a prospective cohort study

**DOI:** 10.3389/fimmu.2026.1766313

**Published:** 2026-03-19

**Authors:** Lijing Xin, Liang Hong, Sijie Wang, Xiaohua Qian, Yue Li

**Affiliations:** 1 Department of Microbiology, Hongkou District Centers for Disease Control and Prevention (Hongkou District Institute of Health Supervision), Shanghai, China; 2 Shanghai Institute of Major Infectious Disease and Biosafety, and Institutes of Biomedical Sciences, Fudan University, Shanghai, China; 3 Key Laboratory of Medical Molecular Virology of MoE&MoH, Shanghai Medical College, Fudan University, Shanghai, China; 4 Department of Immunization Program, Hongkou District Centers for Disease Control and Prevention (Hongkou District Institute of Health Supervision), Shanghai, China; 5 Department of Integrated Operations Management, Hongkou District Centers for Disease Control and Prevention (Hongkou District Institute of Health Supervision), Shanghai, China

**Keywords:** 13-Valent pneumococcal conjugate vaccine, immunogenicity, infants, safety, *Streptococcus pneumoniae*

## Abstract

**Background:**

*Streptococcus pneumoniae* (*S. pneumoniae*) is a major cause of morbidity and mortality in infants, leading to diseases like pneumonia and otitis media. Although the World Health Organization (WHO) recommends inclusion of pneumococcal conjugate vaccines (PCVs) in national immunization programs, 13-Valent PCV (PCV13) remains optional in China, with suboptimal coverage, especially in urban settings like Shanghai. This study was performed to evaluate the safety, immunogenicity and impact on nasopharyngeal carriage of *S. pneumoniae* of PCV13 in healthy infants in Shanghai.

**Material and methods:**

A prospective cohort study was conducted from 2021 to 2023 in Shanghai. Cohort 1 (n=146) assessed immunogenicity and carriage, with infants receiving a 2 + 1 + 1 PCV13 series or no pneumococcal vaccine. Cohort 2 (n=114) assessed safety. Immunogenicity was assessed through serotype-specific IgG concentrations determined by ELISA, expressed as geometric mean concentrations (GMCs). Pneumococcal carriage was evaluated using nasopharyngeal swabs, with serotyping performed by multiplex real-time PCR. One-way repeated-measures ANOVA to compare the IgG GMCs in vaccinated group at three different time points.

**Results:**

In Cohort 1, 69.86% of vaccinated infants completed the 1-year follow-up. Carriage rates were 5.80% in the vaccinated group versus 6.45% in controls one month after the third dose (P = 1.00), and 3.92% versus 13.21% one year after the fourth dose (P = 0.161). IgG GMCs increased significantly from baseline to one month after the third dose (mean difference: 6.41 μg/mL, 95% CI: 5.43-7.39, P<0.001) and to one year after the fourth dose (mean difference: 12.01 μg/mL, 95% CI: 11.28-12.74, P<0.001). In Cohort 2, six infants reported mild adverse events, but no serious events or deaths were noted.

**Conclusion:**

PCV13 induced strong immunogenicity and showed a favorable safety profile, with a non-significant trend toward reduced pneumococcal carriage. These findings provide real-world evidence from Shanghai and support further evaluation of PCV13 in broader cohorts.

## Introduction

*Streptococcus pneumoniae* (*S. pneumoniae*), a significant pathogenic bacterium, remains a leading cause of morbidity and mortality among infants worldwide (1, 2). Globally, it is estimated to cause more than 318,000 deaths annually (with an uncertainty range of 207,000 to 395,000) among children under six years, with Africa bearing the greatest share of this mortality burden (1). In China, pneumococcal meningitis incidence was recently estimated at 2.10 cases per 100,000 children under five each year, with a case fatality rate of 24.6% (3). In 2020 alone, pneumococcal meningitis accounted for approximately 1,617 cases and 549 deaths in Chinese children under five (3). Recognizing this substantial burden, the World Health Organization (WHO) has recommended the inclusion of pneumococcal conjugate vaccines (PCVs) in all national immunization programs (NIPs) since 2007, citing their effectiveness in reducing invasive pneumococcal disease (IPD), pneumonia, and mortality (4).

The 13-PCV (PCV13; Prevnar 13; Pfizer Inc, New York, NY) was developed to broaden the protection offered by the earlier 7-PCV (PCV7; Prevnar 7; Pfizer), including serotypes that account for a significant portion of pneumococcal diseases (5). In November 2016, the Chinese Food and Drug Administration approved PCV13 for active immunization against IPD such as bacteremic pneumonia, meningitis, septicemia, and bacteremia in infants and children from 6 weeks to 15 months old (6). Multiple clinical trials have established the immunogenicity and safety of PCV13 in infants. In Chinese phase 3 trials, PCV13 demonstrated non-inferior serotype-specific IgG responses compared with PCV7, with robust antibody levels against the six additional serotypes unique to PCV13 (7). A 3 + 1 and 2 + 1 infant series both induced protective antibody concentrations, though slightly lower responses were noted for some serotypes in the 2 + 1 schedule (8). Beyond China, PCV13 has shown a consistent safety profile, with most adverse events being mild and transient, including fever, irritability, and injection-site reactions (9, 10). Unlike many countries where PCVs are included in the national immunization program (EPI), in China PCV13 is provided only on a voluntary, out-of-pocket basis, leading to heterogeneous uptake. Recent estimates suggest that PCV13 uptake in urban centers such as Shanghai has improved gradually, yet remains below 30–40% in many districts, far lower than the levels achieved in countries with government-funded immunization (11). Real-world studies have shown good effectiveness of PCV13 in reducing vaccine-type (VT) disease and carriage (12, 13), but challenges remain, including low coverage, socioeconomic disparities in access, and lack of herd immunity effects in most regions.

While safety and immunogenicity are well documented, data on the impact of PCV13 on nasopharyngeal carriage in China remain scarce. Carriage studies are essential because pneumococcal colonization in the nasopharynx is a prerequisite for transmission and invasive disease. Reductions in VT carriage not only provide direct protection for vaccinated children but also contribute to herd immunity by lowering transmission in the community (14). Several studies have shown that PCV introduction significantly decreases carriage of vaccine serotypes (15, 16); however, the magnitude of this effect varies by region, coverage level, and circulating serotypes. In Shanghai, where PCV13 coverage is suboptimal and where our study coincided with the COVID-19 pandemic, children’s social interactions and pathogen transmission dynamics were profoundly altered. Therefore, evaluating the impact of PCV13 on carriage in this unique context provides critical insights into vaccine performance under real-world conditions and helps inform local immunization policy.

Despite these findings, post-marketing data on PCV13, particularly for infants in urban areas like Shanghai, remain sparse. Therefore, this prospective cohort study was performed to comprehensively evaluate PCV13 immunogenicity, safety, and its effect on nasopharyngeal carriage during the COVID-19 pandemic in Shanghai. These findings provide critical real-world evidence to guide local vaccination policy and support broader consideration of PCV13 inclusion in China’s national immunization program.

## Methods

### Study design

This was a prospective, non-randomized, descriptive cohort study, which was conducted during the COVID-19 pandemic period, from January 2021 to August 2023, across six community health centers in the Hongkou District of Shanghai. Eligible participants were stratified into two groups: Cohort 1 (n=146), this 1:1 matched prospective cohort was designed to assess the immunogenicity and impact on nasopharyngeal carriage of *S. pneumoniae* of PCV13 in healthy infants. In this cohort, infants aged 42 to 77 days at the time of enrollment who received PCV13 at 2, 4, 6 at 12 months were assigned into the vaccination group, and the control group comprised infants residing in the same neighborhood, sharing the same sex, not differing in age by more than one month, and who had not been administered any vaccine containing pneumococcal components. Cohort 2 (n=114), this cohort was designed to evaluate the safety of vaccination with the PCV13, and infants in the cohort received at least one dose of the PCV13 between 6 weeks and 15 months of age.

Infants in vaccination group were excluded from the study if they met any of the following criteria: (1) Hypersensitivity to the components of the vaccine and to antibiotics; (2) Acute illness, immunodeficiency or suppression, acute exacerbation of a chronic disease; (3) A serious chronic disease caused by *S. pneumoniae* including congenital anomalies or neurological disease or history of seizures; (4) Prior treatment with blood products or gamma proteins, or participation in another clinical trial. The study protocol was approved by the Ethics Committee of the Hongkou Center for Disease Control and Prevention (Grant number: SHHKCDCP-2020-02). The informed consent was obtained from parents or their legally authorized representatives (LAR).

### Study vaccine and administration

PCV13 (Prevenar 13, Wyeth Vaccines) includes polysaccharides from the seven serotypes found in PCV7 as well as serotypes 1, 3, 5, 6A, 7F, and 19A. The vaccine is delivered in 0.5-mL single-dose syringes, with each serotype represented by 2.2 µg, except for serotype 6B, which is present at 4.4 µg. The formulation also contains 0.125 mg of aluminum, as aluminum phosphate, per 0.5-mL dose. These vaccines are preservative-free and must be stored between 2-8°C. As per local medical guidelines, PCV13 was administered alongside other routine pediatric vaccines. Vaccines were administered by trained vaccinators at community immunization clinics in Hongkou District. PCV13 was administered intramuscularly into the anterolateral thigh (for infants <12 months) or the deltoid muscle (for infants ≥nf months).

### Study objectives

The primary objective of this study was to evaluate differences in nasopharyngeal carriage rates between the vaccinated and control groups. The secondary objective focused on immunogenicity, aiming to demonstrate enhanced immune responses to PCV13 following the completion of the three- and four-dose regimens, as compared to pre-vaccination levels. Additionally, the study assessed the safety profile of PCV13 by monitoring the incidence of local and systemic reactions, as well as adverse events (AEs).

### Sample size

Sample sizes were determined using a two-sample comparison of proportions. At the time of study design, local data on VT carriage were unavailable. Therefore, the overall nasopharyngeal carriage rate among infants under two years in Shanghai (approximately 50%) (17) was used as a proxy reference. Assuming a 50% relative reduction after vaccination, with a two-sided significance level (α) of 0.05 and a power (1-β) of 80%, the required sample size was calculated as 59 per group using PASS software (version 11.0). To compensate for a potential 10% dropout rate, the final target sample size was set at 65 participants per group.

### Nasopharyngeal carriage

**Figure 1:**
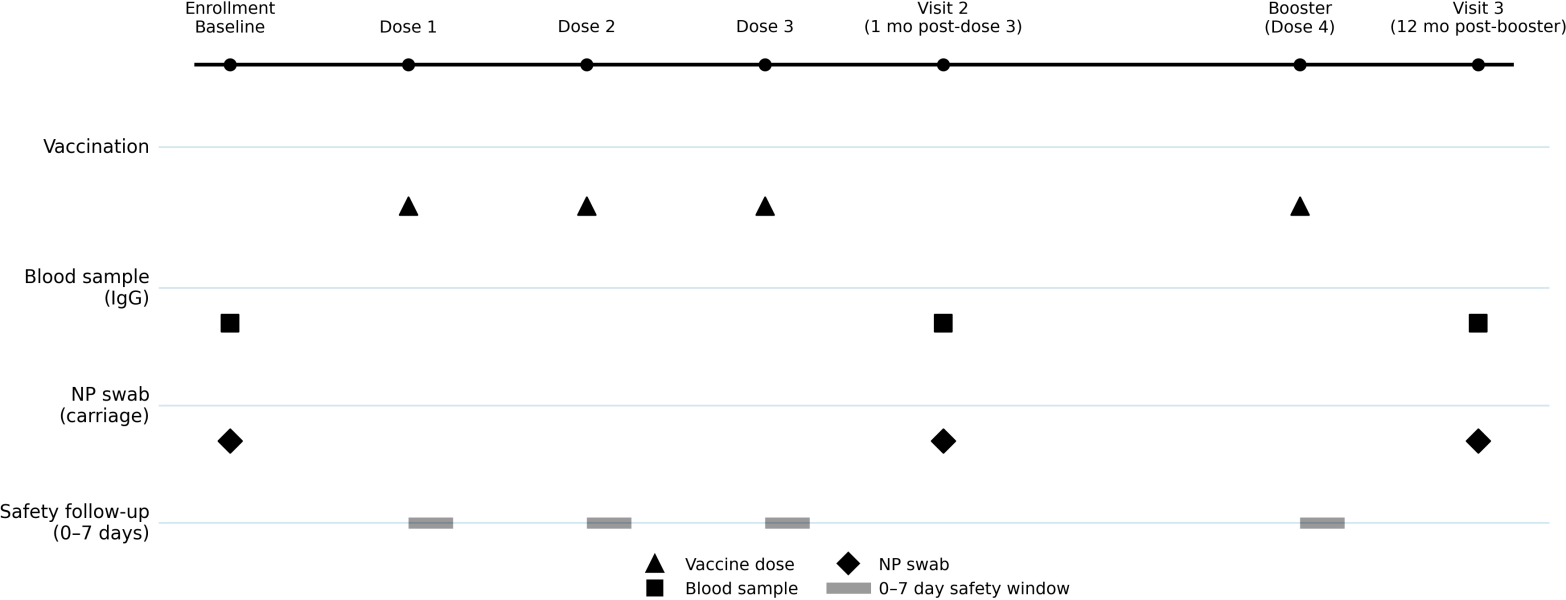
Study design and timeline of vaccination, sample collection, and follow-up. The schematic summarizes the vaccination schedule (primary series and booster), biospecimen collection time points (blood for IgG and nasopharyngeal swabs for carriage), and safety follow-up windows (0–7 days after each dose). Baseline sampling was performed prior to the first dose; post–dose 3 sampling was conducted at 1 month; and long-term sampling was performed 12 months after the booster dose.

All participants in both the vaccinated and control groups are scheduled for nasopharyngeal swabs sampling at predetermined time points: at cohort enrollment, one month after the third dose, and one year after the fourth dose (**Figure 1**). The control group will be collected at corresponding times. After collection, samples will be sent to Hongkou CDC for serotyping. Nasopharyngeal swabs were obtained using sterile, flexible flocked swabs by trained pediatricians and immediately placed in skim milk–tryptone–glucose–glycerol (STGG) medium. Detection and serotyping of *S. pneumoniae* were performed following CDC guidelines (18), using a Multiplex Real-Time polymerase chain reaction (PCR) Diagnostic Kit for Serotyping of *S. pneumoniae* (Beijing Applied Biological Technologies Co., Ltd; Lot No. T202311054). Following blood collection, serum was separated by centrifugation. Throughout the study period, trained staff from community health service centers equipped with cold-chain transport ensured proper sample delivery. All specimens were transported monthly to the Hongkou District CDC laboratory and stored at -80°C until analysis, in accordance with World Health Organization (WHO) guidelines for the collection, transport, and handling of research specimens.

### Immunogenicity evaluations

Blood samples were collected from infants in the vaccinated group at three time points: before vaccination, one month after the third dose and one year after the fourth dose (**Figure 1**). Immunoglobulin G (IgG) levels were measured by enzyme-linked immunosorbent assay (ELISA) according to standardized protocols (18). IgG levels were measured using the Human Streptococcus pneumoniae IgG antibody (SP IgG) ELISA Kit, manufactured by Shanghai Keshun Science and Technology Co., Ltd. (Lot No. 20230501).

### Safety evaluations

Safety assessments were conducted among vaccinated infants in cohort 2.To identify the main types and proportions of AEs occurring in infants following administration of PCV13 through a combination of active monitoring and passive observation. After vaccination, the study population will be provided with diary cards by their parents or guardians to record information on solicited AEs and unsolicited AEs from Day 0 to Day 7, and unsolicited AEs from Day 8 to the next follow-up visit. Parents or guardians will be contacted by telephone on Days 8 through 10 post-vaccination to collect safety information. Serious AEs will be documented and recorded throughout the study period. The observation period extends for one month post-vaccination.

### Missing data and sensitivity analyses

Attrition at the 2-year follow-up was addressed using a prespecified framework based on both Missing-at-Random (MAR) and Missing-Not-at-Random (MNAR) assumptions (19, 20). First, dropout proportions and reasons (moving out of Hongkou/Shanghai, parental withdrawal, and age ineligibility) were summarized by study arm, and baseline characteristics were compared between completers and non-completers. Under the MAR assumption, we performed multiple imputation (21) by chained equations for the binary VT-carriage outcome using a logistic model and including study arm, age, sex, and dropout reason as predictors. Estimates were combined using Rubin’s rules.

To evaluate departures from MAR, we performed a set of MNAR analyses: (i) extreme-case imputation (best-case and worst-case scenarios); (ii) δ-adjusted pattern-mixture models, in which the log-odds of VT carriage for dropouts were shifted by prespecified δ values according to dropout reason.

For each scenario, we re-estimated the risk ratio (RR) and risk difference (RD) between arms using generalized linear models with robust standard errors. Robustness of findings was evaluated based on whether the direction and magnitude of estimates remained consistent across sensitivity analyses.

### Statistical analysis

The primary outcome of this study was the nasopharyngeal carriage of *S. pneumoniae*. Carriage rates were compared between groups using the χ2 test or Fisher’s exact tests where appropriate, utilizing SPSS 20.0 software (IBM Corp., Armonk, NY, USA). One-way repeated-measures analysis of variance (ANOVA) with a Bonferroni *post hoc* test was applied to compare the IgG GMCs in vaccinated group at three time points. Repeated-measures analyses were conducted on complete cases, including only participants with available data at all relevant time points. Participants with missing outcome measurements at any time point were excluded from the primary repeated-measures analysis. Mauchly’s sphericity test was performed to determine whether a violation of sphericity occurred, and the Greenhouse–Geisser correction (when epsilon (ϵ)<0.75) or Huyuh-Feldt correction (when epsilon (ϵ)>0.75) was adopted when the sphericity was violated (22). All P values are two-sided, and significance was defined using a threshold of 0.05.

## Results

### Study subjects

**Figure 2:**
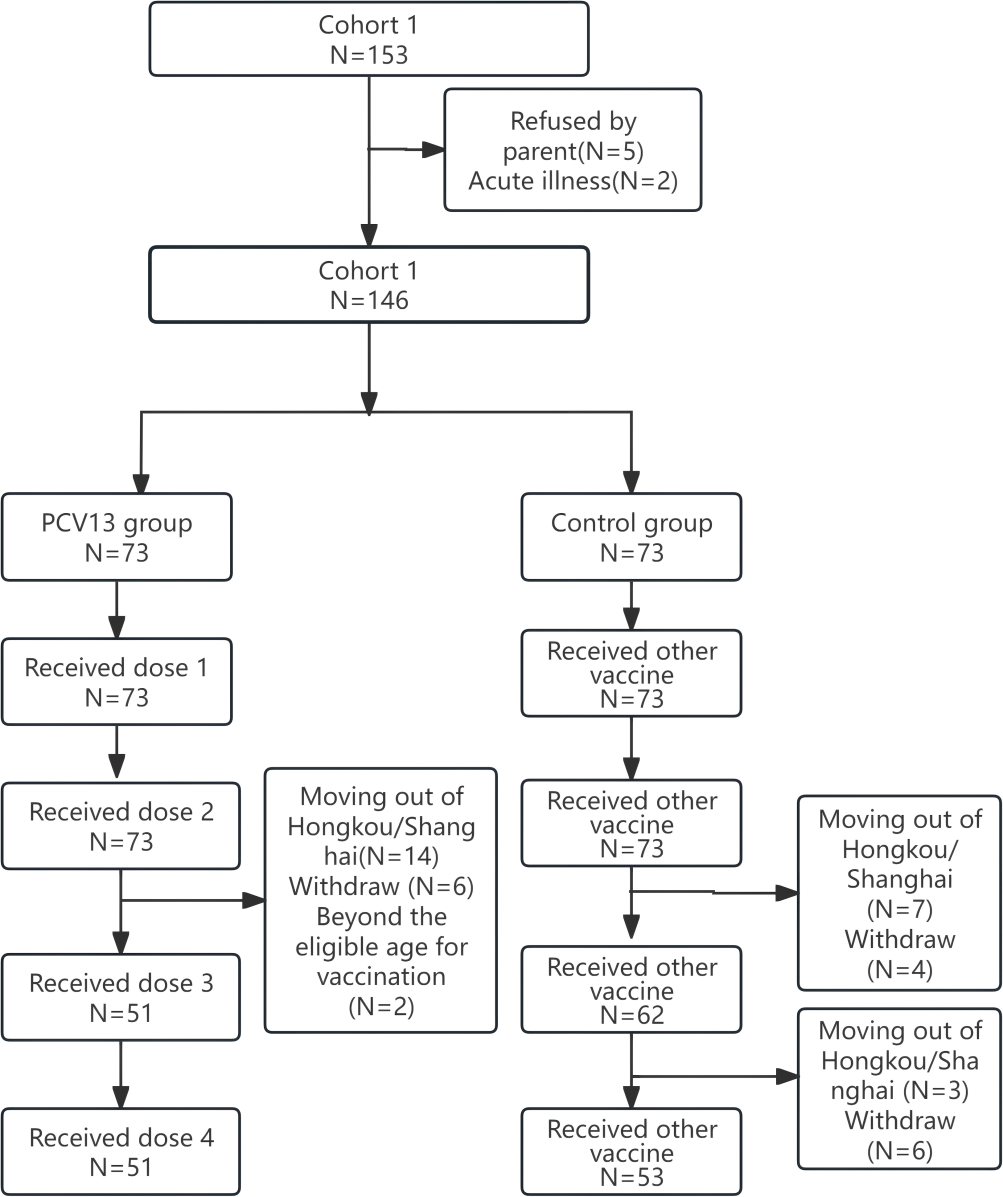
Participant flow diagram for Cohort 1.

In Cohort 1, 153 infants were initially screened. Of these, 5 were excluded due to parental refusal and 2 due to acute illness, resulting in a final enrollment of 146 infants. In the vaccination group, all 73 participants completed the first blood draws, but only 51 (69.83%) completed the second and third blood draws. The main reasons for this shortfall included participants moving out of Hongkou District or Shanghai, reluctance to receive the third dose of the vaccine, aging beyond the eligible age for vaccination, or withdrawing from the study (**Figure 2**).

For nasopharyngeal swab collection, 73 (100%), 69 (94.52%), and 51 (69.83%) participants in the vaccination group completed the first, second, and third swabs, respectively. In the control group, 73 (100%), 62 (84.93%), and 53 (72.60%) participants completed the three swabs, respectively (**Figure 2**).

**Figure 3:**
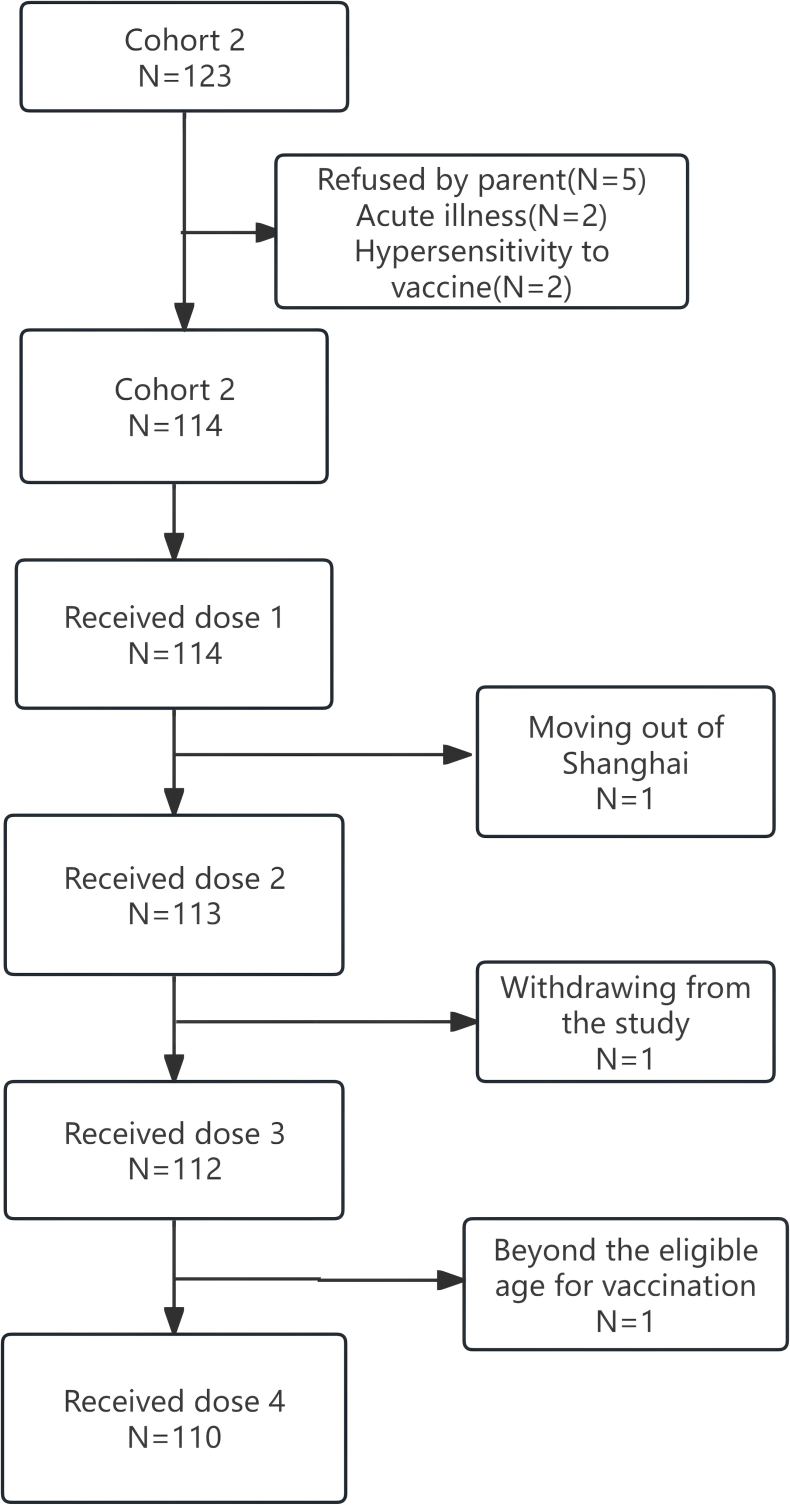
Participant flow diagram for Cohort 2.

In Cohort 2, a total of 123 infants were screened for inclusion in Cohort 2. Among them, 5 were excluded because their parents declined participation, 2 due to acute illness, and 2 owing to hypersensitivity to the vaccine. Ultimately, 114 infants were enrolled. Of these, 114, 113, 112, and 110 infants completed the first, second, third, and fourth doses of the vaccine, respectively (**Figure 3**).

**Table 1** summarizes the baseline individual-level demographic and household characteristics by cohort and study group. Infant demographic characteristics, including sex, birth weight, mode of delivery, Apgar score, and breastfeeding status, were comparable between the PCV13-vaccinated and control groups in Cohort 1 (all P > 0.05).

**Table 1:** Baseline individual-level demographic and household characteristics by cohort and study group.

Characteristic	Cohort 1: PCV13 (n=73)	Cohort 1: Control (n=73)	P value	Cohort 2: PCV13 (n=114)
Infant demographic characteristics				
Male sex	40 (54.79)	41 (56.16)	0.860	62 (54.39)
Birth weight >2.5 kg	69 (94.52)	70 (95.89)	0.650	111 (97.37)
Cesarean delivery	71 (97.26)	69 (94.52)	0.400	98 (85.96)
Apgar score >7 at birth	71 (97.26)	70 (95.89)	0.650	112 (98.25)
Breastfeeding	40 (54.79)	44 (60.27)	0.503	86 (75.44)
Household and socioeconomic characteristics				
≤2 generations in household	67 (91.78)	63 (86.30)	0.290	97 (85.09)
Living area <50 m² per capita	35 (47.95)	42 (57.53)	0.005	42 (36.84)
Monthly household income <20,000 CNY	33 (45.21)	42 (57.53)	0.136	44 (38.60)
Presence of childcare worker at home	18 (24.66)	7 (9.59)	0.016	14 (12.28)
Household health-related exposures				
Household smoking exposure	6 (8.22)	5 (6.85)	0.750	8 (7.02)
Household members with chronic diseases	17 (23.29)	22 (30.14)	0.350	31 (27.19)
Presence of siblings <6 years	12 (16.44)	16 (21.92)	0.400	39 (34.21)
Recent respiratory-related exposures (past 3 months)				
Household respiratory symptoms	4 (5.48)	8 (10.96)	0.230	14 (12.28)
Household outpatient visit	11 (15.07)	13 (17.81)	0.660	18 (15.79)
Household antibiotic use	11 (15.07)	13 (17.81)	0.660	18 (15.79)
Child respiratory symptoms	7 (9.59)	13 (17.81)	0.150	22 (19.30)
Child antibiotic use	7 (9.59)	13 (17.81)	0.150	22 (19.30)

Values are presented as n (%).P values were calculated using Pearson’s chi-square test or Fisher’s exact test, as appropriate.

P values refer to comparisons between PCV13 and Control groups in Cohort 1 only.

Percentages for Cohort 2 were calculated using n=114 as the denominator.

Two-sided P < 0.05 was considered statistically significant.

Most household and socioeconomic characteristics were also similar between groups. However, a significantly higher proportion of participants in the control group resided in households with a living area <50 m² per capita compared with the PCV13-vaccinated group (57.53% vs. 47.95%, P = 0.005). In addition, the presence of a childcare worker at home was more frequent in the PCV13-vaccinated group than in the control group (24.66% vs. 9.59%, P = 0.016). No other significant differences were observed across baseline variables.

### Nasopharyngeal carriage

At enrollment, nasopharyngeal carriage of *S. pneumoniae* was absent in both the vaccinated and control groups (**Table 2**). One month after the third dose of PCV13, the carriage rates were 5.80% (4/69) in the vaccination group and 6.45% (4/62) in the control group, with no significant difference between the two (P = 1.00). One year after the fourth dose of PCV13, carriage rates were 3.92% (2/51) in the vaccination group and 13.21% (7/53) in the control group, but this difference was not statistically significant (P = 0.161). VT carriage in the vaccination group decreased from 5.80% to 3.92%, representing a 1.88% absolute (approximately 32% relative) reduction, which is consistent with the reported 30-40% vaccine efficacy against colonization in previous studies. In contrast, carriage in the control group showed a slight increase, possibly reflecting the absence of vaccination.

**Table 2:** Nasopharyngeal pneumococcal carriage by study group, and time point.

Time point	PCV13(n/N,%)	Control(n/N,%)	Risk difference (95%CI)	P value
Baseline	0/73(0.00)	0/73(0.00)	0.00% (-4.00%, 4.00%)	1.00
Post-dose 3(1 mo)	4/69(5.80)	4/62(6.45)	-0.65% (-0.75%, -0.56%)	1.00
Post-dose 4(12 mo)	2/51(3.92)	7/53(13.21)	-9.29% (-9.46%, -9.12%)	0.161

Data are presented as number of positive cases over total assessed participants (n/N, %). Risk differences were calculated as PCV13-vaccinated minus control group proportions at each time point, with corresponding 95% confidence intervals (CIs). P values were derived using Pearson’s chi-square test or Fisher’s exact test, as appropriate. Analyses were based on available cases at each time point. Two-sided p values <0.05 were considered statistically significant.

Among the seventeen strains identified, the distribution was as follows: 6 strains (35.29%) were serotype 19F, 3 strains (17.65%) were 6B/6D, 3 strains (17.65%) were 6A/6B, 2 strains (11.76%) were 23A, and 3 strains were nontypeable pneumococci (17.65%).

### Immunogenicity to PCV13 after the infant series

To assess whether the IgG GMCs significantly improved over time, multiple measurements at three different time points were analyzed. Mauchly’s sphericity test indicated that the assumption of sphericity was violated for the IgG GMCs (P = 0.019). Consequently, the Huynh-Feldt correction was applied (ϵ=0.899). The corrected results revealed significant differences in IgG GMCs across the three time points [F (1.799, 6.090)=336.253, P< 0.001] (**Table 3**).

**Table 3:** Mauchly’s Test of Sphericity and corrected tests.

Source	Mauchly’s test of sphericity	Corrected tests						
	P	Method	Epsilon (ϵ)	df	Error	F	P	Partial eta squared
IgG GMCs	0.019	Huynh-Feldt	0.899	1.799	6.090	336.253	< 0.001	0.871

Mauchly’s test was performed to assess the assumption of sphericity for repeated-measures analysis. As the sphericity assumption was violated (p = 0.019), the Huynh–Feldt correction was applied. Corrected degrees of freedom (df), error term, F statistic, corresponding p value, and partial eta squared (η²) are presented. Two-sided p values <0.05 were considered statistically significant. IgG, immunoglobulin G; GMCs, geometric mean concentrations.

**Figure 4:**
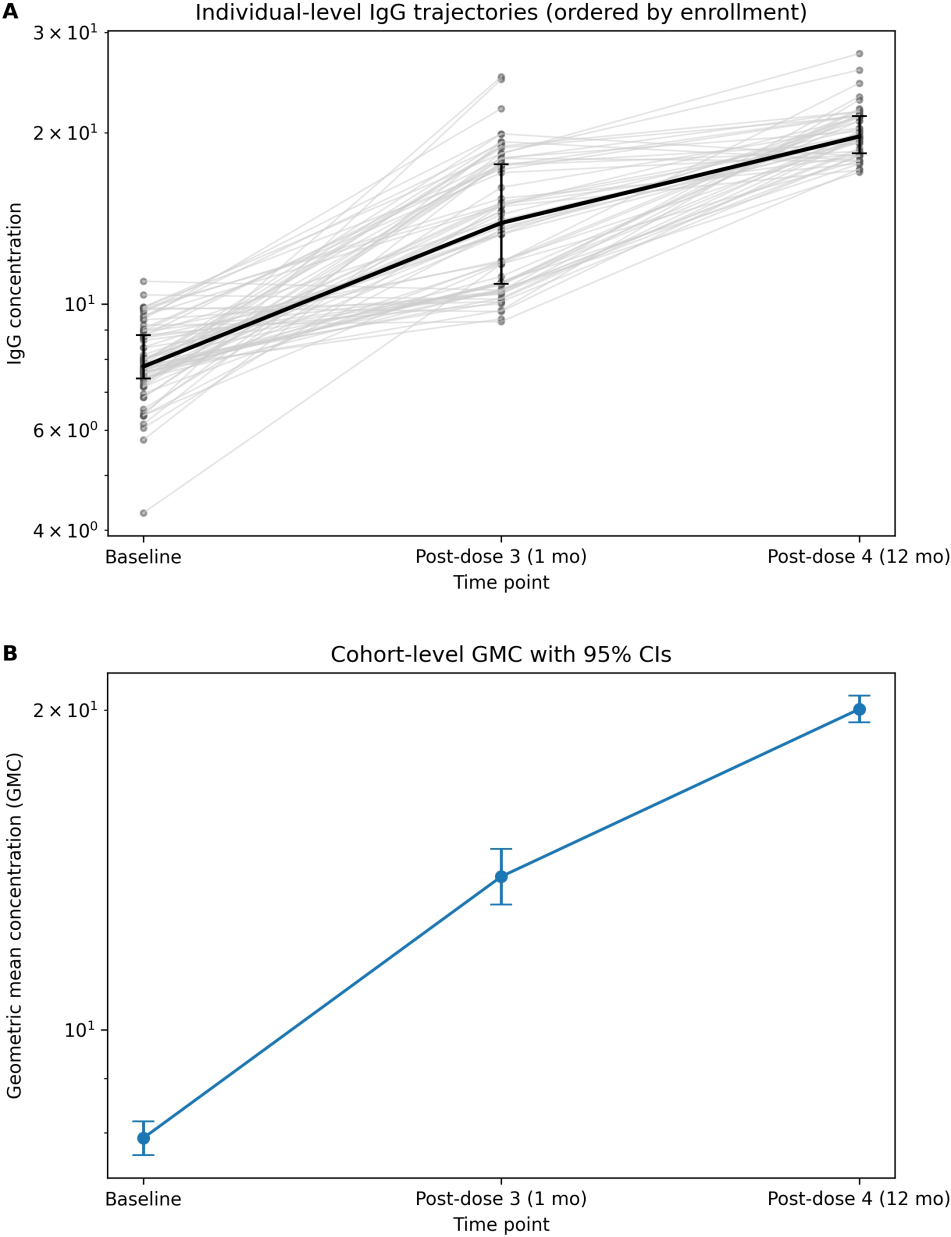
Individual-level and cohort-level serotype-specific IgG responses over time. **(A)** Individual IgG trajectories at baseline, 1 month after the third dose, and 1 year after the booster dose. Thin grey lines represent participants (ordered by enrollment); the thick black line indicates the median with interquartile range (IQR). **(B)** Cohort-level geometric mean concentrations (GMCs) with 95% confidence intervals at each time point, calculated on the log scale and back-transformed. Analytic sample sizes were n=73 at baseline, n=73 at post-dose 3, and n=51 at post-dose 4.

Pairwise comparison analysis demonstrated significant changes in IgG GMCs between the time points. Specifically, the IgG GMCs increased by 6.412 μg/mL one month after the third dose compared to baseline. One year after the fourth dose, IgG GMCs showed an increase of 12.012 μg/mL from baseline (**Figure 4**). Additionally, IgG GMCs increased by 6.349 μg/mL one year after the fourth dose compared to one month after the third dose (**Table 4**).

**Table 4:** Pairwise comparisons of the IgG GMCs in vaccinated group at three time points.

Pairwise comparisons	Mean difference	Std. Error	P	95 CI%	
Baseline VS. 1 month after dose 3	-6.412	0.502	< 0.001	-7.389	-5.434
Baseline VS. 1 year after dose 4	-12.012	0.363	< 0.001	-12.742	-11.282
1 month after dose 3 VS. 1 year after dose 4	-6.349	0.510	< 0.001	-7.373	-5.325

Analyses were based on complete-case data. Participants with missing measurements at any time point were excluded from the repeated-measures ANOVA. GMC, geometric mean concentrations.

### Safety

In cohort 2, 114 infants received the first dose of PCV13. During the 7-day follow-up period, 6 infants (3 males and 3 females) reported fever with temperatures ranging from 37.7°C to 39.0°C (**Table 5**). The fever developed approximately 10 to 17 hours after vaccination and resolved the following day after physical cooling and adequate hydration. Additionally, four infants exhibited redness and swelling at the injection site, and one child showed signs of irritability. No AEs were reported during follow-up visits for the second through fourth doses of vaccine.

**Table 5:** Solicited and unsolicited adverse events following PCV13 vaccination.

Event	Dose 1 n/N(%)	Dose 2-4 n/N(%)
Fever	6/114(5.26)	None
Redness and swelling	4/114(3.51)	None
Irritability	1/114(0.88)	None

Adverse events were recorded within 7 days after each vaccination dose. Data are presented as number of events over total vaccinated participants (n/N, %). No adverse events were reported following doses 2–4 during the monitoring period. No serious adverse events were observed.

### Sensitivity analyses for dropout

Detailed results of sensitivity analysis for dropout are provided in **Table 6**. At the 2-year visit, attrition occurred in 22/73 (30.1%) children in the PCV13 group and 20/73 (27.4%) in the control group, mainly due to moving out of Hongkou/Shanghai or withdrawal by parents. The distribution of dropout reasons was similar between arms.

**Table 6:** Sensitivity analyses assessing the impact of dropout on VT nasopharyngeal carriage estimates.

Scenario	Missing-data assumption	VT carriage (%), PCV13 vs. Control	RR	RD
Complete-case (primary analysis)	Only observed data included	3.9% vs. 13.2%	0.30 (95% CI: 0.06–1.36)	–9.3% (95%CI: –19.8%- 1.3%)
Multiple Imputation (MAR)	Missing at random; imputes based on group, demographics, and dropout reason	4.1% vs. 13.7%	0.30 (95% CI: 0.09–1.05)	–9.6%
MNAR – Best-case	All missing in PCV13 = non-carrier; all missing in Control = carrier	2.7% vs. 37.0%	0.07	–34.2%
MNAR – Worst-case	All missing in PCV13 = carrier; all missing in Control = non-carrier	32.9% vs. 9.6%	3.43	+23.3%
MNAR–Moderate deviation (risk ×2 among vaccinated dropouts)	Vaccinated dropouts assumed to have twice the observed risk; Control unchanged	5.1% vs. 13.2%	0.39	–8.1%

Sensitivity analyses were conducted to evaluate the robustness of VT carriage estimates under different missing-data assumptions. The primary analysis was based on complete-case data. Multiple imputation under the missing-at-random (MAR) assumption was performed using group assignment, demographic characteristics, and dropout reason as predictors.

Missing-not-at-random (MNAR) scenarios included best-case, worst-case, and moderate deviation assumptions to explore extreme and intermediate departures from MAR. VT carriage proportions are presented for PCV13 versus control groups. Risk ratios (RR) and risk differences (RD) are reported where applicable, with 95% confidence intervals (CIs) provided when estimable. MAR, missing at random; MNAR, missing not at random; VT, vaccine-type; RR, risk ratio; RD, risk difference.

In the complete-case analysis, VT carriage was 3.9% (2/51) in the PCV13 group and 13.2% (7/53) in the control group (RR = 0.30, 95% CI 0.06–1.36; RD = –9.3%, 95% CI –19.8% to 1.3%). Under a MAR assumption using multiple imputation, the overall VT carriage was 4.1% versus 13.7%, yielding an almost identical estimate (RR = 0.30, 95% CI 0.09–1.05).

Under MNAR scenarios, the best-case and worst-case imputations produced RRs of 0.07 and 3.43, respectively, representing theoretical lower and upper bounds. More moderate assumptions (e.g. two-fold higher risk among vaccinated dropouts) yielded RRs below 0.4. Overall, these sensitivity analyses suggest that while statistical power is limited, the direction of the effect estimate is robust to plausible missing-data mechanisms.

## Discussion

This cohort study evaluated the safety, immunogenicity, and impact on nasopharyngeal carriage of *S. pneumoniae* following administration of the PCV13 in healthy infants in Shanghai. The results indicated that the nasopharyngeal carriage rates of *S. pneumoniae* were not significantly different between the vaccination and control groups one month after the third dose and one year after fourth dose of PCV13. Additionally, IgG GMCs increased significantly one month after the third and one year after fourth dose as compared to baseline. The IgG GMCs also increased significantly at one year after fourth dose as compared to one month after third dose. PCV13 was well tolerated, with the only AE reported being fever occurring on the day after the first dose. These findings suggest that PCV13 is well tolerated and provides robust immunogenicity for the included serotypes in healthy Chinese infants.

S. *pneumoniae* is a leading cause of bacterial pneumonia and meningitis in infants under five in China (23, 24). A recent systematic review and meta-analysis, covering studies from 1980 to 2022, aimed to provide a comprehensive understanding of the pneumococcal meningitis during periods of low PCV coverage in China (3). This review reported an estimated incidence of pneumococcal meningitis of 2.10 cases per 100,000 infants under 5 years each year, with a pooled case fatality rate of 24.59% (3). In 2020, it was estimated that there were 1617.16 cases of pneumococcal meningitis and 548.86 deaths among children under five in China (3). The review also found that *S. pneumoniae* was responsible for 22.05% of confirmed bacterial meningitis cases (3). These findings underscore the critical need for comprehensive surveillance and the inclusion of PCVs in the national immunization schedule to reduce the burden of pneumococcal diseases in Chinese children.

To our knowledge, this is the first cohort study to evaluate the safety, immunogenicity and impact on nasopharyngeal carriage of *S. pneumoniae* after PCV13 administration in China. In cohort 1, we observed that the nasopharyngeal carriage rates of *S. pneumoniae* in the vaccinated group one year after the fourth dose and one month after the third dose were lower than those in the control group; however, these differences were not significant. Although the lack of statistical significance, the observed 32% relative reduction in VT carriage among vaccinated children is consistent with previously reported PCV13 efficacy against colonization (30–40%) (25–27). The predominance of VT serotypes (6B, 19F, 6A) further supports that the observed decline may reflect vaccine impact (28). Given the small sample size and lower-than-expected baseline VT carriage, these findings should be interpreted with caution.

Our findings should be interpreted in light of differences from other domestic and international studies. For example, a cross-sectional survey conducted in Hainan Province, China, reported a substantially higher overall pneumococcal carriage rate (30.4%) among children aged 0–59 months, with VT serotypes accounting for 60.9% of isolates (29). In that study, VT carriage was significantly lower among vaccinated children than among unvaccinated children (41.9% vs. 62.7%), suggesting a measurable effect of PCV13 vaccination on nasopharyngeal colonization. Similarly, a recent cross-sectional study by Du et al. (30) investigated nasopharyngeal carriage of *S. pneumoniae* among 4,911 healthy children aged 30 days to <60 months in Beijing and Shenzhen between 2018 and 2021. The overall carriage rate was 13.1%, and 36.7% of isolates were VT serotypes. Carriage was significantly lower among PCV7/PCV13-vaccinated children compared with unvaccinated peers (10.7% vs. 14.9%, P<0.05) (30). Notably, in Beijing, the authors observed a marked decline in carriage over time—particularly during the COVID-19 pandemic—suggesting potential effects of both vaccination and non-pharmaceutical interventions on transmission dynamics.

Internationally, studies from Spain and Mongolia have also documented substantial reductions in VT pneumococcal carriage following PCV13 introduction (31–34).These investigations were conducted under routine immunization programs with relatively high vaccine coverage (approximately 70–80%) and included sampling of children around the post-booster age (12–24 months), which may have amplified the observed impact on VT carriage. Surveillance and carriage-study data from England indicate that under sustained high PCV13 coverage, VT carriage and VT-related IPD incidence have declined markedly, consistent with strong direct and indirect effects (35, 36).

In Cohort 1, we observed a significant improvement in IgG GMCs from baseline to one month after the third dose and one year after the fourth dose. Our findings align with those of previous studies (37). Chu K, et al. performed a randomized, open-label, phase 3 trial in Chinese infants and children under 6 years of age, demonstrating that serotype-specific IgG GMCs increased substantially, ranging from 0.01 µg/mL (serotypes 4 and 18C) to 0.38 µg/mL (serotype 5) at baseline, and rising to 1.19 (serotype 3) to 10.89 (serotype 14) µg/mL one month after the vaccination series. Notably, ≥95.5% of participants achieved IgG concentrations ≥0.35 µg/mL for each serotypes at one month post-vaccination (37). However, contrasting findings were reported in a study conducted in Kuwait between 2010 and 2019, where the authors found that PCV13 did not appear to provide substantial protection against invasive disease caused by the six PCV13 non-PCV7 serotypes (1, 3, 5, 6A, 7F, and 19A) (38). In that study, no significant decrease in invasive disease due to the non-PCV7 serotypes of PCV13 was observed during period III (August 2013–July 2019) or when comparing combined periods II (August 2010–July 2013) and III with period I (August 2003–July 2006). Despite this, significant reductions were noted for four of the six serotypes and the overall serotypes included in PCV7, as well as the total serotypes included in PCV13. The reduction in total PCV13 serotypes was mainly attributed to the serotypes covered by PCV7 (38).

The authors of the Kuwait study suggest that the apparent low efficacy of PCV13 against these specific serotypes could be influenced by several factors, including surveillance and data limitations, potential misclassification of strains, and statistical power issues, rather than an inherent lack of vaccine effectiveness. First, they note the potential unreliability of their passive surveillance system, which may obscure true vaccine effects due to inconsistencies in reporting and isolate collection. Second, their analysis may be biased by focusing on the relative prevalence of vaccine serotypes compared to non-vaccine serotypes (NVTs), as vaccination could increase NVTs and lead to a perceived reduction in vaccine serotypes without a true reduction in their absolute numbers. Third, they highlight the lack of comprehensive data on vaccine coverage, which may limit the accuracy of their conclusions. Fourth, they raise the possibility that the increase in non-typeable strains after vaccination may be due to technical problems, such as improper handling, which could misclassify these strains as non-typeable. Finally, they suggest that the small number of cases involving the PNP serotypes may have led to an underpowered statistical analysis, weakening the robustness of their findings.

The safety profile of PCV13 observed in this study is in line with global safety data. The most common AEs were mild and transient, such as injection site redness, swelling, and pain, along with systemic reactions like fever and irritability. No serious AEs related to the vaccine were reported, corroborating the well-established safety of PCV13 (8, 37, 39, 40). Moreover, no unsolicited AEs were reported in the present study during the follow-up period. This is consistent with findings from one domestic clinical trial conducted in Chinese infants, which reported no unsolicited or serious vaccine-related events following PCV13 administration (7, 8). These results collectively support the favorable safety profile of PCV13 in the Chinese population. In contrast, some international PCV13 safety studies and meta-analyses have included reports of unsolicited AEs, including occasional upper respiratory symptoms, although these are generally mild and not attributed to vaccine causality. For example, in a Canadian infant cohort, Vanderkooi et al. evaluated PCV13 given with routine pediatric vaccines and included unsolicited AE monitoring (41). A meta-analysis by Ruiz-Aragón et al. combining multiple PCV13 trials also summarized the spectrum of solicited and unsolicited AEs, concluding an overall tolerable safety profile in infants (42). Although these studies do not uniformly emphasize respiratory unsolicited AEs, their existence underscores that such events are captured in regulated trials, and the absence of such events in our cohort further supports PCV13’s favorable safety profile.

From a clinical and public health perspective, our findings provide important insights for vaccination strategies in Shanghai and similar urban settings. Despite limited statistical significance, the observed trend of reduced vaccine-type carriage, combined with strong immunogenicity and favorable safety, supports the clinical utility of PCV13. These results highlight the importance of improving vaccine uptake, reducing economic barriers to access, and considering inclusion of PCV13 in the national immunization program. For clinicians, the evidence reinforces PCV13 as a safe and effective option for individual protection, while for policymakers, it underscores the broader potential to reduce pneumococcal transmission and disease burden through higher coverage rates.

Several limitations of this study should be acknowledged. Firstly, the study population was limited to children in Hongkou district, Shanghai, and the findings may not be generalizable to other populations in China or other countries. Secondly, sample size was determined using overall carriage rates as a proxy due to the lack of local VT carriage data at the time of study design. As a result, the statistical power may have been limited to detect modest differences in VT carriage between groups. Thirdly, although minor baseline imbalances were observed in household living area and childcare worker presence, these variables were not directly related to the primary immunogenicity outcomes. Therefore, no covariate adjustment was performed. Lastly, the study was conducted during the COVID-19 pandemic, which could have influenced the outcomes. Consequently, the results should be interpreted within the context of the pandemic, and further research is needed to evaluate PCV13’s effectiveness in the post-pandemic period.

In conclusion, this study demonstrates that PCV13 is both immunogenic and safe in healthy infants, with evidence suggesting a potential reduction in nasopharyngeal carriage of *S. pneumoniae*. These results reinforce the importance of including PCV13 in routine immunization programs for infants, particularly in regions with a high burden of pneumococcal disease. Further research with larger cohorts and longer follow-up is warranted to fully elucidate the vaccine’s impact on pneumococcal carriage and to optimize strategies for preventing pneumococcal infections in diverse populations.

## Data Availability

The raw data supporting the conclusions of this article will be made available by the authors, without undue reservation.
